# Prevalence and Characteristics of Multisite Musculoskeletal Symptoms among District Hospital Nurses in Haiphong, Vietnam

**DOI:** 10.1155/2020/3254605

**Published:** 2020-05-29

**Authors:** Thanh Hai Nguyen, Duc Luan Hoang, Thi Giang Hoang, Minh Khue Pham, Julie Bodin, Jean Dominique Dewitte, Yves Roquelaure

**Affiliations:** ^1^Faculty of Public Health, Haiphong University of Medicine and Pharmacy, Vietnam; ^2^Univ Angers, CHU Angers, Univ Rennes, Inserm, EHESP, Irset (Institut de Recherche en Santé, Environnement et Travail)-UMR_S 1085, F-49000 Angers, France; ^3^Phu Tho College of Medicine and Pharmacy, Vietnam; ^4^Occupational Health and Environmental Diseases Department, CHRU Morvan-Laboratory for Studies and Research in Sociology (EA3149), University of Western Brittany, Brest, France

## Abstract

**Background:**

Musculoskeletal disorders (MSDs) are commonly observed among workers around the world. These diseases not only affect the health of workers, their quality of life, and their performance, but the effects of such diseases also represent a great burden for the health and social systems. These issues are even more prevalent in developing countries, and nurses are no exception. Many studies worldwide have shown a high prevalence of work-related MSDs in each body position among nurses. However, there are very few studies that have mentioned multisite musculoskeletal symptoms (MMS).

**Objectives:**

To describe the prevalence and characteristics of MMS among district hospital nurses in Haiphong, Vietnam. *Material and Methods*. A cross-sectional study was carried out on 1179 nurses working in all 15 district hospitals using the Modified Nordic Questionnaire at 9 anatomical sites on the body (neck, shoulder/upper arm, elbow/forearm, wrist/hand, upper back, lower back, hip/thigh, knee/lower leg, and ankle/foot). The following main indicators were calculated: the prevalence of musculoskeletal symptoms (MS) (at least 1 of 9 sites), MMS (two or more sites), and widespread musculoskeletal symptoms (WMS) (MS of the upper limb, the lower limb, and the back or the neck).

**Results:**

The prevalence of MS during the past 12 months and symptoms lasting for at least 30 days was 60.6% and 17.2% in men and 77.6% and 21.5% in women, respectively. The lower back, neck, upper back, and shoulder/upper arm were the most common sites affected. In terms of MMS, the prevalence was 37.6% in men and 57.1% in women during the past 12 months while 8.6% of men and 11.3% of women reported that symptoms lasted for at least 30 days. The prevalence of MMS tended to increase with age, seniority, having a history of musculoskeletal diseases, and in nurses working in district hospitals located in urban areas. Nearly 90% of MMS concerned two or three anatomical regions during the past 12 months, and almost 80% of MMS lasting at least 30 days involved two or three anatomical regions. The prevalence of WMS was 10.4% in men and 18.6% in women during the past 12 months and 0.9% in men and 2.1% in women lasting at least 30 days.

**Conclusions:**

This study showed the high prevalence of MMS and WMS among nursing staff. Further and more extensive research is needed to improve our understanding of multisite musculoskeletal symptoms and act as the foundation for developing preventive measures for nurses.

## 1. Introduction

The term musculoskeletal disorders (MSDs) is a long-standing and popular concept. It refers to health issues related to the locomotor apparatus such as muscles, tendons, bones, cartilage, ligaments, the vascular and nervous systems, and other soft tissues and joints [1]. Work-related musculoskeletal disorders include all disorders that are caused or aggravated by work and associated working conditions [2]. These disorders are very common in workers all around the world. In Europe, MSDs represent a large part of the health problems suffered by workers. Every year, millions of workers in Europe in a variety of professions are affected by MSDs due to their work [2]. According to the 6th European Working Conditions Survey, MSDs are among the most reported health problem: backache (reported by 43%), muscular pain in the neck or upper limbs (42%), and muscular pain in the hip or lower limbs (29%) [3]. MSDs not only affect workers' health, quality of life, and performance, but their effects also represent a great burden for the health and social systems [4, 5].

Pain at multiple sites is defined as pain that occurs in at least 2 of all anatomical sites surveyed. In similar studies, the prevalence period differed: the preceding 12 months (Yeung et al. 2012 [6], Solidaki et al. [7]), the previous month only (Miranda et al. [8], Coggon et al. [9]), or the preceding 3 months (Haukka et al. [10]). Additionally, the commonly studied form of musculoskeletal pain at multiple sites was chronic widespread pain (CWP) which is one of the two diagnostic criteria of fibromyalgia in 1990 of the American College of Rheumatology (ARC) [11]. In this report, pain is considered chronic and widespread where pain lasts more than 3 months and has the following characteristics: pain in the left and right side of the body, pain above and below the waist, and axial skeletal pain (cervical spine or anterior chest or thoracic spine or low back) must be present. Fibromyalgia's diagnostic criteria were later adjusted to better adapt to the clinical reality in 2010 [12] and most recently in 2018 [13]. However, the concept of CWP is not dissimilar to the aggravating and wide level of pain. Nevertheless, it appears that the prevalence of CWP in the population was found to be lower than pains experienced in multiple sites out with the defined CWP distribution [14]. Within the limited scope of this study, we only addressed multisite musculoskeletal pain or multisite musculoskeletal symptoms (MMS). In this respect, MSDs at multiple sites are more prevalent than single-site disorders due to the interactions and interrelatedness of affected sites. In a study by Coggon et al. among 12,410 adults (aged between 20 and 59), MSDs (mainly chronic pain) at multisite were more common than single site issues [9]. Therefore, it is more pertinent to evaluate and describe the characteristics of MMS.

In terms of careers, MSDs are the most frequent occupational health problem among health care workers, especially in nurses [15]. A recent systematic review of Soylar and Ozer found that the prevalence of MSDs varied between 33.0% and 88.0% and the work-related MSDs were associated with multiple factors such as demographic characteristics of nurses as well as physical, psychological, and organizational factors [16]. However, all studies were only considered and analyzed in relation to simple MSDs (in each anatomical site), but not to multiple MSDs or MMS.

In Vietnam, research on occupational health is still limited. In the population of nurses, there was only a few studies regarding issues related to occupational exposures (exposure to blood and secretions, occupational tuberculosis, or radiation …) [17] or mental health problems (stress, anxiety, and depression) [18]. For MSDs, there were two existing studies published to date: the first one by Kieu et al. in 2016, conducted in over 300 nurses working at a regional hospital, having demonstrated a high prevalence of MSDs (81%) during the past 12 months [19]; and the second one is our study which was carried out on all nurses working at all district hospitals in Haiphong city where the general prevalence was 74.7 [20]. However, both studies have evaluated the general prevalence of MSDs and of each anatomical site but did not go into MMS, a more meaningful value than as mentioned above.

For all the arguments above, we would like to take advantage of the available data from previous research to further analyze MMS, thereby improving the understanding of the specific characteristics and interactions between anatomical sites in MMS among district hospital nurses.

## 2. Materials and Methods

### 2.1. Study Design

A cross-sectional study was carried out from January to June 2017 to determine the prevalence and describe the characteristics of multisite musculoskeletal symptoms among nurses working in district hospitals of Haiphong, Vietnam.

### 2.2. Sample Size and Recruitment of Study Subjects

Study subjects included all nurses working in each of the 15 district hospitals in Haiphong city in Vietnam who gave their consent to participate in this study. A total of 1179/1279 (participation rate: 92.2%) nurses were surveyed (100 nurses who refused to participate or were not present at the time of the interview were excluded).

### 2.3. Research Instrument and Data Collection

Data were collected through two questionnaires. The questionnaires were used by our researcher for direct interviews with the participants. Each interview ranged from 30 to 45 minutes.

(1) A sociodemographic questionnaire: this questionnaire is used to collect some general information of participants such as age, gender, height, weight, history of musculoskeletal diseases, and some information about their professional situation: position, seniority, shift work…

(2) Modified Nordic Questionnaire: this questionnaire was based on the Standardized Nordic Questionnaire that was developed by Kuorinka et al. in 1987 [21]. This questionnaire was divided into two main parts: the first part evaluates general health problems related to the musculoskeletal system at 9 different positions on the body (neck, shoulder/upper arm, elbow/forearm, wrist/hand, upper back, lower back, hip/thigh, knee/lower leg, and ankle/foot) during the last 12 months and within the last 7 days; the second one assesses the specific problem of MSDs in each position (including characteristics of the total duration of musculoskeletal problems in the past 12 months) as well as the impact the MSDs have on the professional and personal lives of the respondent.

### 2.4. Definitions of Evaluation Criteria

Musculoskeletal symptoms (MS) have been treated by dividing them into 2 categories: during the past 12 months and lasting at least 30 days during the past 12 months.

MMS are defined by the presence of MS declared by the subject on two or more anatomical sites among the nine anatomical sites studied. For bilateral anatomical sites, MS were classed as present if they were reported on either one or both sides of the body.

The 3 anatomical regions studied were as follows:

- Axial: upper back and lower back
- Upper limb: shoulder/upper arm, elbow/forearm, wrist/hand, and neck
- Lower limb: hip/thigh, knee/lower leg, and ankle/foot

Definition of “widespread musculoskeletal symptoms” based on the American College of Rheumatology (ARC): musculoskeletal symptoms of the upper limb (with neck), the lower limb, and the back (upper or lower back).

### 2.5. Statistical Analysis

Data were analyzed using SPSS software (v19.0). Prevalence (number, percentage, 95% confidence interval) of MS in each site and of MMS according to sex, age group, seniority, position type, shift work, and history of musculoskeletal diseases was calculated. The following statistical tests were used: a Chi-squared test or Fisher's exact test when comparing two percentages, the LLA (Linear-by-linear Association) test for a linear trend when comparing more than two percentages across levels of age and seniority groups and an ANOVA test when comparing at least three means were used. All analyses were performed separately in men and women. The level of significance was set at a *p* value of less than 0.05, and the number and frequency were not presented when *n* ≤ 5.

### 2.6. Ethics

The study was approved by the Haiphong University of Medicine and Pharmacy, Institutional Review Board and authorized by the Haiphong Department of Health to implement the study at its district hospitals. Informed consent was obtained for all nurses that participated in the survey.

## 3. Results

### 3.1. Sociodemographic Characteristics

The study involved a total of 1179 nurses working in all of 15 district public hospitals in Haiphong city, and 81.3% of them are women. The average age of female and male nurses was 32.6 years (SD = 7.2) and 32.3 years (SD = 9.9), respectively. In terms of position type, nurses were divided into two types of jobs: administrative (only in charge of managing patients' records, not directly caring for patients) (9.4%) and clinical (directly caring for patients) (90.6%). Most nurses have worked for a long time in their current position: 94.7% more than 1 year, 65.4% more than 5 years, 31% more than 10 years, and 15.5% more than 15 years. Additionally, 68.3% of them had to work night shifts (on duty). Lastly, 11.2% of the nurses surveyed had experienced musculoskeletal diseases during their life at some stage.

### 3.2. Prevalence of Musculoskeletal Symptoms (MS)

The prevalence of nurses with MS in at least 1 anatomical in the past 12 months in men was 60.6% (54.2-67.0) and 77.6% (75.0-80.2) in women (*p* < 0.001, Table 1). A total of 38 male (17.2% (12.2–22.2)) and 206 female (21.5% (18.9–24.1)) nurses reported that they had at least one MS lasting at least 30 days during the past 12 months (*p* = 0.154). The four anatomical sites with the highest prevalence of MS were lower back, neck, upper back, and shoulder/upper arm, both during the past 12 months and lasting at least 30 days. In detail, during the past 12 months, the anatomical site with the highest prevalence of MS in men was the neck with 36.2% (29.9-42.5), followed by the lower back with 28.5% (22.5-34.5). In the same period, the highest prevalence sites for women were the lower back with 47.9% (44.7-51.1) and the neck with 45.2% (42.0-48.4). Overall, the prevalence of MS at each anatomical site in women was higher than in men and these differences were statistically significant (*p* < 0.05), except at the elbow/forearm, hip/thigh, and ankle/foot. Similarly, the anatomical sites with the highest prevalence of MS lasting at least 30 days in the past 12 months in men and women are the lower back and neck. The corresponding prevalence was 7.7% (4.2-11.2) and 7.2% (3.8-10.6) in men and 12.2% (10.1-14.3) and 7.2% (5.6-8.8) in women. In general, the prevalence of MS lasting at least 30 days in women was also higher than in men; however, these differences are not statistically significant (*p* > 0.05) (Table 1).

### 3.3. Prevalence of Multisite Musculoskeletal Symptoms (MMS)

The prevalence of MMS in women was significantly higher than in men during the past 12 months (57.1% versus 37.6%, *p* < 0.001). The prevalence of MMS during the past 12 months tends to increase significantly with seniority in women only. Moreover, the prevalence in the group of nurses with a history of musculoskeletal disease is significantly higher than nurses who do not previously had MMS, 70.8% versus 33.5% in men (*p* < 0.001) and 77.8% compared to 54.5% in women (*p* < 0.001). Finally, the MMS prevalence of women working in hospitals located in urban areas was significantly higher than women working in hospitals located in rural areas (63.9% versus 53.6%, *p* = 0.002) (Table 2).

There was a significant association between MMS lasting at least 30 days in the past 12 months with the age groups and seniority in women. The prevalence of the age group from 19 to 29 was the lowest (7.8%) while the group of 50 to 60 years old displayed the highest rate (27.6%) (*p* < 0.01). Similarly, in terms of seniority, the prevalence of MMS increased from 4.8% in the group of less than 1 year to 20.4% in more than 15 years (*p* < 0.001). In both sexes, nurses with a history of musculoskeletal disease had MMS that persisted for at least 30 days longer than those who did not have previous MMS issues. This rate was 29.2% compared with 6.1% in men (*p* < 0.01) and 33.3% compared with 8.5% in women (*p* < 0.001) (Table 3).

**Figure 1:**
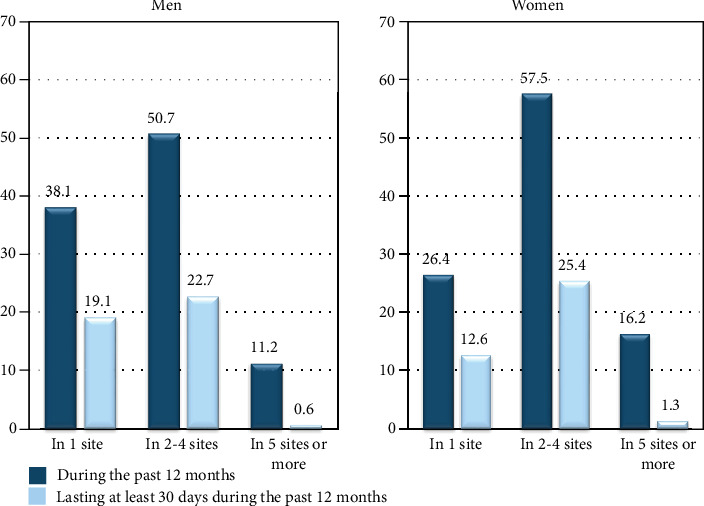
Distribution of musculoskeletal symptoms according to the number of anatomical sites with musculoskeletal symptoms during the past 12 months as observed in the 134 men and the 743 women studied.

According to the number of symptoms, the percentage of MS in 2 to 4 sites was more significant than in 1 site or in 5 sites or more in both sexes. This was 50.7% during the past 12 months, and nearly half of them (22.7%) lasted for at least 30 days for men. The corresponding percentage in women was 57.5% and 25.4% (Figure 1).

**Figure 2:**
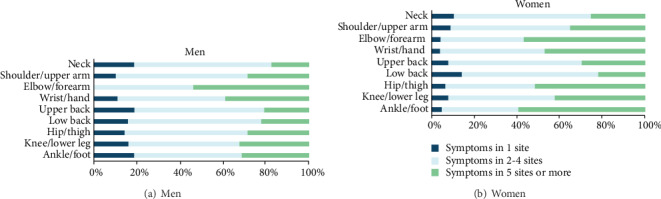
Distribution of musculoskeletal symptoms during the past 12 months according to the number of anatomical sites.

**Figure 3:**
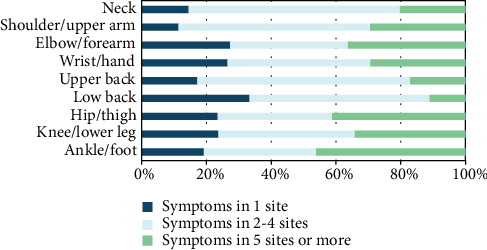
Distribution of musculoskeletal symptoms lasting at least 30 days during the past 12 months according to the number of anatomical sites in women.

When considering the distribution of MS according to the number of sites by combination in each site with other sites, the prevalence of MS during the past 12 months at 2 to 4 sites was 3 to 12 times higher than the prevalence at a single site for nurses who reported MS at any given anatomical site. The only exception to this was in the elbow/forearm site in men. There were no cases of MS in this site in isolation, but there was a case of MS in the elbow/forearm and another anatomical site (Figures 2(a) and 2(b)). Furthermore, for women, the prevalence of MS lasting at least 30 days at 2 to 4 sites ranged from ca. 40% to ca. 70% while prevalence in 5 sites or more ranged from ca. 10% to ca. 45% approximately (Figure 3). The prevalence rate for men was not presented due to the low number of men having MS lasting at least 30 days during the past 12 months.

### 3.4. Prevalence of MS in Various Anatomical Regions

**Figure 4:**
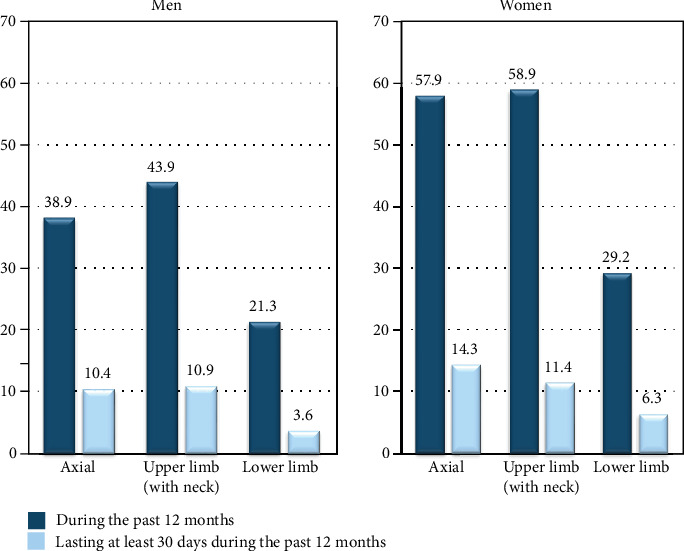
Prevalence of musculoskeletal symptoms in various anatomical regions among men and women.

MS appeared mainly in the axial and upper limb (with neck) regions during the past 12 months. Prevalence rates were 38.9% and 43.9% for men and 57.9% and 58.9% for women, respectively (Figure 4). According to the number of anatomical regions, more than one quarter of nurses reported the presence of MS in a single region during the past 12 months (27.6% in both sexes), mainly in the upper limb region in men (47.5%) and in the axial region among women (46.8%). Two regions were affected in nearly one quarter of men (22.6%) and nearly one third in women (31.3%). This primarily involved a combination of the axial and upper limb regions with 70% in men and 74% in women. Furthermore, 10.4% of men and 18.6% of women reported that the MS involved all three anatomical regions (axial, upper limb, and lower limb) (Table 4). Lastly, more than 10% of nurses declared MS lasting at least 30 days during the past 12 months in only one region and ca. 6% in two regions with a predominance of the axial and the upper limb regions (from 65% to 85%) (Table 5).

Generally, nearly 90% of multisite MS concerned two or three anatomical regions (88% for men and 87.4% for women) during the past 12 months. Almost 80% of multisite MS lasting at least 30 days involved two or three anatomical regions (79% for men and 74.1% for women).

### 3.5. Prevalence of Widespread Musculoskeletal Symptoms (WMS)

During the past 12 months, 10.4% of men and 18.6% of women displayed WMS (MS in all of three regions). Only 0.9% in men and 2.1% in women had WMS lasting at least 30 days during the past 12 months (Tables 4 and 5).

## 4. Discussion

### 4.1. Main Result

This study was conducted on 1179 nurses working at all of the district hospitals in Haiphong city to better understand the prevalence and characteristics of MMS. The main results are as follows:

- The prevalence of MMS during the past 12 months was considerable with more than half of all nurses being affected, with a higher rate in women than in men (57.1% versus 37.6%). In addition, nearly 90% of MMS concerned two or three anatomical regions
- A significant number of nurses indicated that they had MMS lasting at least 30 days (8.6% in men, 11.3% in women) and almost 80% of MMS involved more than one anatomical region
- The percentage of MS in 2 to 4 sites was greater than in 1 site or in 5 sites or more in both sexes

### 4.2. Prevalence of MS

Generally, the prevalence of MS among nurses in our study was high with a rate of 74.4% over a one-year time period (60.6% for men and 77.6% in women). It is easy to see that this result is quite similar to the results in many other studies. Without doubt, there have been many studies and reports all over the world which have highlighted the high prevalence of work-related MSDs in health workers, especially on nurses, within the study period of 12 months. Ellapen and Narsigan conducted a systematic review of work-related MSDs among nurses in 2014, and the results showed that the mean work-related MSDs among the analyzed publications were 71.85%. Furthermore, the most vulnerable anatomical sites were the lower back, neck, and shoulders [22], which are exactly the same as our research results. Another systematic review of Soylar and Ozer in 2018 showed that the prevalence of MSDs varied between 33.0% and 88.0% and the most commonly affected body regions were lower back, shoulders, neck, knees, and wrists/hands [16]. Other recent studies in Asia, especially neighbouring countries in the same region, have shown similar results, such as in Malaysia (73.1%, most commonly affected sites were the neck and shoulders) [23], in China (79.5%, most commonly affected sites were the waist, neck, and shoulder) [24], in Lebanon (73.1%) [25], and a little higher in Thailand (83.9%, most commonly affected sites were lower back, shoulder, and knee) [26]. Some countries in Europe and Africa reported higher prevalence rates: 88.6% in Portugal [27], 98% in Greece [28], in Zimbabwe [29], and in Egypt [30, 31].

In the United States, the prevalence of MSDs in nurses was lower than the results in our study as well as other studies. For example, research by Zhang et al. found a prevalence of 47.4% in nurses [32]. In this respect, many prevention programs have been implemented in the US a long time ago to prevent and reduce MSDs in nurses [33]. In addition, the conditions and working environment of nurses in the US are also better than in Vietnam [34], which can limit the exposure to hazards in the nurse's working environment.

The prevalence of MSDs in women is higher than in men in our study for both MS and MMS, and the mechanisms explaining these differences between sexes are poorly understood. However, some studies report a higher prevalence of musculoskeletal pain in women than in men in the general population. These differences may partly be explained by differences in vulnerability or risk factors for musculoskeletal pain such as obesity and older age—both having affecting women only—and pain catastrophizing, which was more often associated with musculoskeletal pain among men than among women [35, 36].

### 4.3. Prevalence of MMS

It should be emphasized that some studies have mentioned MMS in the general working population [8, 37, 38]; however, the number of MMS studies on nurses is very limited and nursing is a career group with many risk factors with MSDs [16]. Therefore, in comparison to other studies, it is understandable that there will be differences due to different subjects and research methods.

Results of MMS prevalence during the past 12 months and lasting at least 30 days all revealed a higher rate in women than men in nurses with a history of musculoskeletal disease compared to those without prior MS issues. This prevalence tends to increase with age and seniority, and more than half of nurses reported that they had the MS in 2 to 4 anatomical sites. This is clearly consistent with the evidence in similar studies [37, 39]. A study by Neupane et al. revealed that over 52% of the 1348 health care sector employees in selected occupational groups (paramedics, allied health, patient orderlies, food service assistants, cleaners, and allied health personnel including nurses) reported pain in multiple body sites and 19% reported pain in one site [40]. Another study from Sembajwe et al. in the USA also presented similar results: 50.3% (of 1572 patient care workers from two large hospitals in the greater Boston area—70.5% staff nurses) had two pain sites or more and only 23.4% had one pain site [41]. In a French general working population, Parot-Schinkel et al. have shown slightly higher MMS prevalence, 63.2% of men and 68.3% for women during the past 12 months [37]. This difference can be explained by differences in study populations, and it is possible that in Parot-Schinkel et al.'s study, there are groups of people with a higher prevalence of MMS compared to nurses (shift or office workers for example). This may also be evident in Solidaki et al.'s research with two-thirds of the study sample (39.7% of nurses) reporting pain in 2 body sites or more during the past 12 months [7]. This difference may be accounted for by the different populations targeted (office workers and postal clerks in addition to nurses) and by the number of sites studied (6 sites versus 9 sites in our study).

The above differences are also reflected in the results of the MS prevalence in various anatomical regions among men and women. The prevalence of MS in each anatomical region (axial, upper limb, and lower limb) in Parot-Schinkel et al.'s study was higher than that in our studies [37]. However, both studies have one thing in common: that the prevalence of MS in axial and upper limb (with neck) regions was generally higher than in the lower limb region for both sexes. These results reaffirmed that MSDs mainly occur in the upper limbs and back of nurses and in the general population. This result is also shown in the relationship between the number of anatomical regions and the symptoms of MSDs during the past 12 months and lasting at least 30 days (Tables 4 and 5). In nurses displaying musculoskeletal symptoms, the prevalence of MS was higher in cases where symptoms appeared in the axial and upper limb regions, regardless of sex and the number of regions affected. These results are almost identical to the results of Parot-Schinkel et al.'s study in that the axial and upper limb regions are the two main regions affected by MS [37].

When considering the distribution of MS during the past 12 months according to the number of anatomical sites, the prevalence of MS during the past 12 months at 2 to 4 sites was far greater than at a single site for nurses who reported MS at any given anatomical site. This result is quite similar to the results of Parot-Schinkel et al.'s study in that the prevalence of MS affecting 2 to 4 anatomical sites was 3 to 12 times more common than the prevalence of MS affecting only one site in workers who reported MS at any given anatomical site [37].

The final result that we have learned in this study is the prevalence of widespread MS. The definition of widespread MS that we used is the same one that Picavet and Schouten [42] and Parot-Schinkel et al. [37] used (musculoskeletal symptoms in the upper limb or the neck, the lower limb, and the back). We found that this definition is almost similar to one of the two diagnostic criteria of fibromyalgia of ARC in 1990 [11] which has been used in many studies [14, 43–45]. The prevalence of widespread MS during the past 12 months in our study was slightly higher than in Cho et al.'s study among the general population in Korea [14] (with 16.2% and 5.5% in females and males, respectively) but was lower than that in the Parot-Schinkel et al.'s study among the French population with 26.8% in men and 27.7% in women (prevalence of MS in all of three regions during the past 12 months) [37]. The reason for this difference is that there were differences in the study subjects as well as the definition used for widespread pain. Additionally, two recent systematic review studies that have used the definition of chronic widespread pain (defined as widespread pain lasting more than 3 months) were shown the prevalence in general population from 10.6% to 11.8% [45] and from 8.0% to 11.2% [44]. This shows that these rates are slightly lower than the prevalence of widespread MS in our study. Through this evidence, it can be seen that the prevalence of MMS as well as the widespread MS in nurses is similar and high as in the general population. It also means that more research is needed on MMS and chronic widespread pain among nurses to better understand the prevalence and key features of MMS. This could be the basis for future intervention studies more efficient about MMS on nurses.

### 4.4. Weaknesses and Strengths of the Study

One of the strengths of this study is that it was the first study in Vietnam to investigate MSDs in nurses, especially regarding the characteristics of MMS. In the past, there were a few studies in Vietnam assessing MSDs on workers in some other occupations [46, 47] and only one in nurses [19] but the second one did not mention MMS and their characteristics. Therefore, this study will be a basis to be able to open further studies on MSDs on nurses (for building risk and consequence models as well as preventive measures for MSDs). In addition, although all study sites were district hospitals, the scale and characteristics of work were very diverse with hospitals located in the city center (urban region) and hospitals located in both suburban and rural areas as well. The functions and work of nurses at district hospital level are the same; however, in district hospitals located in urban regions, the work intensity and density of nurses are higher than in hospitals located in rural areas. This is explained by the population density in urban areas being much higher than in rural areas, so the demand for public health care is also higher. This is also reflected in the results that the prevalence of MMS during the past 12 months among female nurses working in hospitals located in urban areas is higher than that in rural areas. Therefore, with the large and varied sample size, it can be assumed that the research sample was highly representative of all nurses in Haiphong in particular and in Vietnam in general. Finally, this study used the Nordic questionnaire which has been used extensively in many countries around the world for studying MSDs in various subject groups and particularly among nurses [48].

However, this study has several limitations. Like all other studies that used the Nordic questionnaire to evaluate MSDs, the respondents' responses were subjective and may have recalled information bias when they were asked about MS in the past 12 months. In addition, this questionnaire has not been officially validated into the Vietnamese language. Nevertheless, the investigators have been well trained and have tried to coordinate these events carefully to limit this bias. In the exclusion criteria, our study was not able to exclude other persistent musculoskeletal diseases from the beginning of the survey; this may affect the assessment of current symptoms of MSDs such as congenital spine disorders, trauma, pain caused by surgery, or other diseases. Another point to emphasize is the lack of clinical criteria (examination and testing) to correctly diagnose symptoms or syndromes of MSDs. In addition, there was a gender imbalance among participants in this study. The percentage of women is 81.3% which is approximately 4.5 times higher than the number of men (18.7%). However, this difference is derived from the characteristics of nursing work. This is a profession that requires skill in work, attentiveness, and dedication to take care of patients. Many statistics around the world have also shown that nursing has always been female dominated [49]. Thus, providing separate results and analysis for men and women appears to be reasonable and acceptable in this study. Lastly, this cross-sectional study cannot provide any information about the chronology and course of the symptoms described. This is an area for development that would improve future studies on MSDs in nurses in particular and in general workers.

## 5. Conclusion

This study highlights a high prevalence of multisite musculoskeletal symptoms and widespread musculoskeletal symptoms among nursing staff. Further research is needed to better understand these multisite musculoskeletal symptoms and provide a foundation for implementing preventive measures for nurses.

## Figures and Tables

**Table 1:** Prevalence of musculoskeletal symptoms among men and women.

	Men (*n* = 221)			Women (*n* = 958)			*p* value
	*n*	%	95% CI	*n*	%	95% CI	
During the past 12 months							
At least one anatomical site	134	60.6	54.2-67.0	743	77.6	75.0-80.2	<0.001
Neck	80	36.2	29.9-42.5	433	45.2	42.0-48.4	0.015
Shoulder/upper arm	49	22.2	16.7-27.7	293	30.6	27.7-33.5	0.013
Elbow/forearm	13	5.9	2.8-9.0	95	9.9	8.0-11.8	0.061
Wrist/hand	18	8.1	4.5-11.7	176	18.4	15.9-20.9	<0.001
Upper back	53	24.0	18.4-29.6	320	33.4	30.4-36.4	0.007
Lower back	63	28.5	22.5-34.5	459	47.9	44.7-51.1	<0.001
Hip/thigh	7	3.2	0.9-5.5	62	6.5	4.9-8.1	0.059
Knee/lower leg	31	14.0	9.4-18.6	205	21.4	18.8-24.0	0.014
Ankle/foot	16	7.2	3.8-10.6	84	8.8	7.0-10.6	0.462
Lasting at least 30 days in the past 12 months							
At least one anatomical site	38	17.2	12.2-22.2	206	21.5	18.9-24.1	0.154
Neck	16	7.2	3.8-10.6	69	7.2	5.6-8.8	0.985
Shoulder/upper arm	11	5	2.1-7.9	44	4.6	3.3-5.9	0.807
Elbow/forearm	a			22	2.3	1.4-3.2	
Wrist/hand	a			34	3.5	2.3-4.7	
Upper back	10	4.5	1.8-7.2	64	6.7	5.1-8.3	0.234
Lower back	17	7.7	4.2-11.2	117	12.2	10.1-14.3	0.056
Hip/thigh	a			17	1.8	1.0-2.6	
Knee/lower leg	6	2.7	0.6-4.8	38	4.0	2.8-5.2	0.376
Ankle/foot	a			26	2.7	1.7-3.7	

^a^Not presented, *n* ≤ 5.

**Table 2:** Prevalence of multisite musculoskeletal symptoms during the past 12 months.

Multisite MS	Men (*n* = 221)			*p* value	Women (*n* = 958)			*p* value
	*n*	%	95% CI		*n*	%	95% CI	
Gender (Chi-square test)	83	37.6	31.2-44.0	—	547	57.1	54.0-60.2	—
Age group (years)				0.513^∗∗^				0.054^∗^
19-29	40	34.2	27.9-40.5		192	53.3	50.1-56.5	
30-39	27	43.5	37.0-50.0		248	58.4	55.3-61.5	
40-49	7	31.8	25.7-37.9		89	61.8	58.7-64.9	
50-60	9	45.0	38.4-51.6		18	62.1	59.0-65.2	
Seniority				0.299^∗∗^				0.010^∗^
Less than 1 year	6	28.6	22.6-34.6		21	50.0	46.8-53.2	
1-5 years	30	34.9	28.6-41.2		137	52.9	49.7-56.1	
5-10 years	23	40.4	33.9-46.9		195	56.0	52.9-59.1	
10-15 years	12	57.1	50.6-63.6		101	62.3	59.2-65.4	
More than 15 years	12	33.3	27.1-39.5		93	63.3	60.2-66.4	
Position type				0.282^∗∗^				0.299^∗∗^
Administrative	4	25.0	19.3-30.7		59	62.1	59.0-65.2	
Clinical	79	38.5	32.1-44.9		488	56.5	53.4-59.6	
Shift work				0.701^∗∗^				0.843^∗∗^
Yes	60	36.8	30.4-43.2		368	57.3	54.2-60.4	
No	23	39.7	33.2-46.2		179	56.6	53.5-59.7	
History of musculoskeletal diseases				<0.001^∗∗^				<0.001^∗∗^
Yes	17	70.8	64.8-76.8		84	77.8	75.2-80.4	
No	66	33.5	27.3-39.7		463	54.5	51.3-57.7	
Location of hospital				0.289^∗∗^				0.002^∗∗^
Urban region	17	31.5	25.4-37.6		209	63.9	60.9-66.9	
Rural region	66	39.5	33.1-45.9		338	53.6	50.4-56.8	

^∗^Test for linear trend–LLA. ^∗∗^Chi-square test.

**Table 3:** Prevalence of multisite musculoskeletal symptoms lasting at least 30 days in the past 12 months.

Multisite MS	Men (*n* = 221)			*p* value	Women (*n* = 958)			*p* value
	*n*	%	95% CI		*n*	%	95% CI	
Gender (Chi-square test)	19	8.6	4.9-12.3	—	108	11.3	9.3-13.3	—
Age group (years)				0.055^∗^				<0.001^∗^
19-29	6	5.1	2.2-8.0		28	7.8	6.1-9.5	
30-39	7	11.3	7.1-15.5		49	11.5	9.5-13.5	
40-49	3	13.6	9.1-18.1		23	16.0	13.7-18.3	
50-60	3	15.0	10.3-19.7		8	27.6	24.8-30.4	
Seniority				0.079^∗∗^				<0.001^∗^
Less than 1 year	2	9.5	5.6-13.4		2	4.8	3.4-6.2	
1-5 years	3	3.5	1.1-5.9		17	6.6	5.0-8.2	
5-10 years	5	8.8	5.1-12.5		36	10.3	8.4-12.2	
10-15 years	4	19.0	13.8-24.2		23	14.2	12.0-16.4	
More than 15 years	5	13.9	9.3-18.5		30	20.4	17.8-23.0	
Position type				0.634^∗∗^				0.434^∗∗^
Administrative	2	12.5	8.1-16.9		13	13.7	11.5-15.9	
Clinical	17	8.3	4.7-11.9		95	11.0	9.0-13.0	
Shift work				0.108^∗∗^				0.569^∗∗^
Yes	11	6.7	3.4-10.0		75	11.7	9.7-13.7	
No	8	13.8	9.3-18.3		33	10.4	8.5-12.3	
History of musculoskeletal diseases				0.002^∗∗^				<0.001^∗∗^
Yes	7	29.2	23.2-35.2		36	33.3	30.3-36.3	
No	12	6.1	2.9-9.3		72	8.5	6.7-10.3	
Location of hospital				0.090^∗∗^				0.080^∗∗^
Urban region	8	14.8	10.1-19.5		45	13.8	11.6-16.0	
Rural region	11	6.6	3.3-9.9		63	10.0	8.1-11.9	

^∗^Test for linear trend–LLA. ^∗∗^Chi-square test.

**Table 4:** Number of anatomical regions with musculoskeletal symptoms during the past 12 months.

	Men (*n* = 221)			Women (*n* = 958)		
MS in	*n*	%	95% CI	*n*	%	95% CI
No region	87	39.4	33.0-45.8	215	22.4	19.8-25.0
One region	61	27.6	21.7-33.5	265	27.6	24.8-30.4
Axial	23	10.4	6.4-11.4	124	12.9	10.8-15.0
Upper limb (with neck)	29	13.1	8.7-17.5	116	12.1	10.0-14.2
Lower limb	9	4.1	1.5-6.7	25	2.6	1.6-3.6
Two regions	50	22.6	17.1-28.1	300	31.3	28.4-34.2
Axial and UL	35	15.8	11.0-20.6	223	23.3	20.6-26.0
Axial and LL	5	2.3	0.3-4.3	30	3.1	2.0-4.2
UL and LL	10	4.5	1.8-7.2	47	4.9	3.5-6.3
Three regions	23	10.4	6.4-14.4	178	18.6	16.1-21.1

**Table 5:** Number of anatomical regions with musculoskeletal symptoms lasting at least 30 days during the past 12 months.

	Men (*n* = 221)			Women (*n* = 958)		
MS in	*n*	%	95% CI	*n*	%	95% CI
No region	183	82.8	77.8-87.8	752	78.5	75.9-81.1
One region	23	10.4	6.4-14.4	126	13.2	11.1-15.3
Axial	9	4.1	1.5-6.7	65	6.8	5.2-8.4
Upper limb (with neck)	10	4.5	1.8-7.2	42	4.4	3.1-5.7
Lower limb	a			19	2.0	1.1-2.9
Two regions	13	6.0	2.9-9.1	60	6.3	4.8-7.8
Axial and UL	11	5.0	2.1-7.9	39	4.1	2.8-5.4
Axial and LL	a			13	1.4	0.7-2.1
UL and LL	a			8	0.8	0.3-1.3
Three regions	a	0.9		20	2.1	1.3-2.9

^a^Not presented, *n* ≤ 5.

## Data Availability

The SPSS data used to support the findings of this study are available from the corresponding author upon request.
